# *Parasedimentitalea* *nitratireducens* sp. nov., a Novel Nitrate-Reducing Bacterium Isolated from the Yellow Sea

**DOI:** 10.3390/microorganisms14081706

**Published:** 2026-08-04

**Authors:** Yanjiao Zhang, Xinyu Liu, Hao Yu, Xiuqing Yang

**Affiliations:** Shandong Provincial Key Laboratory of Microbial Resource Exploration and Innovative Utilization, School of Life Sciences, Qingdao Agricultural University, Qingdao 266109, China; zhangyanjiao34@163.com (Y.Z.); 17664422874@163.com (X.L.); yuhaosunshine@163.com (H.Y.)

**Keywords:** *Parasedimentitalea nitratireducens* sp. nov., nitrate reduction, Yellow Sea, marine sediment, polyphasic taxonomy

## Abstract

A Gram-stain-negative, rod-shaped, aerobic strain CY06^T^ was isolated from surface marine sediment sample of the Yellow Sea, China. The strain was positive for oxidase and catalase and could reduce nitrate to nitrite but not further to gaseous nitrogen products. Phylogenetic analysis based on 16S rRNA gene sequences and the up-to-date bacterial core gene (UBCG) sequences revealed that strain CY06^T^ formed a distinct clade within the genus *Parasedimentitalea*. Genomic comparisons, including digital DNA-DNA hybridization (dDDH), average nucleotide identity (ANI) and average amino acid identity (AAI), clearly distinguished strain CY06^T^ from other members of the genus. The morphological, physiological, biochemical and chemotaxonomic data further supported its differentiation from closely related species. Based on the polyphasic evidence, a new species, *Parasedimentitalea nitratireducens* sp. nov., is proposed, with the type strain CY06^T^ (=MCCC 1K08636^T^ = KCTC 72006^T^).

## 1. Introduction

The genus *Parasedimentitalea* belongs to the family *Rhodobacteraceae* within the class *Alphaproteobacteria*. It was first proposed by [1] with the description of *P. marina* from deep-sea water. To date, the genus contains five recognized species: *P. marina* [1], *P. psychrophila* [2], *P. denitrificans*, *P. huanghaiensis* and *P. maritima* [3].

Members of this genus are aerobic and Gram-stain-negative, with C_18:1_ *ω*7*c* and/or C_18:1_ *ω*6*c* as the major fatty acids and Q-10 as the major quinone. All species were isolated from marine environments, including deep-sea water and marine sediments [1,2,3,4,5]. Some species of *Parasedimentitalea*, such as *P. denitrificans*, *P. huanghaiensis* and *P. maritima*, can reduce nitrate and also perform complete denitrification [3,5]. Thus, *Parasedimentitalea* species with denitrification potential may play a role in the nitrogen cycle of marine environments.

In this study, a bacterial strain, designated CY06^T^, was isolated from a surface marine sediment sample. This strain is capable of reducing nitrate to nitrite but is unable to reduce nitrite further or perform denitrification. Based on a polyphasic taxonomic study, this strain is proposed to represent a novel species of the genus *Parasedimentitalea*. Notably, this unique nitrate reduction phenotype suggests possible diversity in nitrate reduction capability within the genus *Parasedimentitalea*.

## 2. Materials and Methods

### 2.1. Type Strains and Culture Conditions

Strain CY06^T^ was isolated from a surface sediment sample collected from the Yellow Sea using a standard dilution-plating method. Unless otherwise stated, the strain was routinely cultured on marine agar 2216 (MA; Difco, Franklin Lakes, NJ, USA) or in marine broth 2216 (MB; Difco) at 20 °C. The reference strains *P. huanghaiensis* MCCC 1K04409^T^, *P. maritima* KCTC 62762^T^, and *P. denitrificans* MCCC 1K08635^T^ were also grown under the same conditions.

### 2.2. 16S rRNA Gene Sequencing and Phylogenetic Analysis

After a single colony of strain CY06^T^ was grown in MB to exponential phase, the cells were harvested by centrifugation (4 °C, 10,000 rpm, 3 min), washed twice with sterile artificial seawater (ASW) [6] and then used for genomic DNA extraction. Genomic DNA was extracted using a bacterial genomic DNA isolation kit (BioTeke, Beijing, China) following the manufacturer’s instructions, except that sterile ddH_2_O was used for DNA elution. The 16S rRNA gene was amplified by PCR with universal primers 27F (5′-AGAGTTTGATCCTGGCTCAG-3′) and 1492R (5′-GGTTACCTTGTTACGACTT-3′) [7] from genomic DNA. The resulting PCR products were purified using an E.Z.N.A. gel extraction kit (Omega Bio-tek, Norcross, GA, USA). The purified PCR products were then ligated into the pMD 19-T vector (Tsingke Biotechnology Co., Ltd., Beijing, China), transformed into *Escherichia coli* DH5α and sequenced by Tsingke (Qingdao, China). The nearly complete 16S rRNA gene sequence was compared with those of validly published species and type strains using the EzBioCloud server [8] (ChunLab, Inc., Seoul, Republic of Korea). Phylogenetic trees were constructed with MEGA version 12.1.2 [9] using maximum-likelihood [10], maximum-parsimony [11] and neighbour-joining [12] methods. The robustness of the tree topologies was assessed by bootstrap analysis with 1000 replicates.

### 2.3. Genome Sequencing and Genome-Based Phylogeny

Genomic DNA of strain CY06^T^ was extracted following the method described above. Purified DNA was assessed for quality and concentration using a NanoDrop One spectrophotometer (Thermo Fisher Scientific, Waltham, MA, USA), and only samples with an OD_260/280_ ratio of 1.8–2.0 were used for genome sequencing. Whole-genome sequencing was performed at BGI (Shenzhen, China) using the Illumina HiSeq 4000 platform. The draft genome sequence was annotated using the NCBI Prokaryotic Genome Annotation Pipeline [13]. The genomic DNA G+C content was calculated from the draft genome sequence deposited in the GenBank database.

Genome-based relatedness indices including digital DNA-DNA hybridization (dDDH), average nucleotide identity (ANI), and average amino acid identity (AAI) were also calculated. The dDDH values were estimated using the Genome-to-Genome Distance Calculator (GGDC) with the recommended formula 2 [14]. ANIb values were calculated using JspeciesWS (https://jspecies.ribohost.com/jspeciesws/, accessed on 6 May 2026). AAI values were calculated using the CompareM software package (version 0.1.1 https://github.com/dparks1134/CompareM, accessed on 6 May 2026), applying a minimum amino acid identity threshold of 40% and a minimum sequence coverage of 50% [15].

The phylogenetic tree was reconstructed using the up-to-date bacterial core gene (UBCG) set, which comprises 92 single-copy core genes [16]. The concatenated alignment of the 92 core genes was generated from genome sequences using UBCG version 3 [16] and was subsequently used as input for phylogenetic tree reconstruction. A maximum-likelihood (ML) tree was constructed using MEGA version 12.1.2 with the General Time Reversible (GTR) model plus gamma-distributed rate variation with invariant sites (GTR+G+I), as determined by the best-fit model selection in MEGA. The robustness of the tree topology was assessed by bootstrap analysis with 100 replicates. *Roseovarius tolerans* EL-172^T^ was used as the outgroup.

### 2.4. Phenotypic and Chemotaxonomic Characterizations

Cellular morphology was examined using a transmission electron microscope (HITACHI HT7700) with cells taken from exponentially growing cultures. Cell motility was examined by light microscopy using wet mounts to observe swimming motility according to the method described by [17]. The presence of flagella was further assessed by transmission electron microscopy following negative staining with 2% (*w*/*v*) phosphotungstic acid and using a commercial flagella staining kit (Qingdao Hope Bio-Technology Co., Ltd., Qingdao, China). Colony morphology was observed following 7 days of incubation on MA at 20 °C. Gram staining was performed according to the standard Gram-staining procedure described by [18]. Growth at different temperatures was tested in MB at 5, 10, 15, 20, 25, 30, 37 and 42 °C. NaCl tolerance was determined in NP broth [19] at 20 °C with NaCl concentrations of 0%, 0.5%, and from 1.0% to 10.0% (in increments of 1.0%, *w*/*v*). The pH range for growth was assessed in NP broth containing 2.0% NaCl and buffered with 50 mM of MES (pH 5.0–6.0), MOPS (pH 6.5–7.0), Tris (pH 7.5–8.5) or CHES (pH 9.0–10.0). Catalase activity was determined by observing bubble production in 3% (*v*/*v*) H_2_O_2_. Oxidase activity was detected using commercial test strips (Hangzhou Tianhe Microorganism Reagent Co., Hangzhou, China) following the manufacturer’s instructions. Hydrolysis of Tween 40, Tween 60 and Tween 80 was tested using MA as the basal medium. Nitrate reduction, denitrification and nitrite reduction were tested in MB medium amended with 0.1% (*w*/*v*) KNO_3_ or 0.01% (*w*/*v*) NaNO_2_, each in tubes containing a small inverted Durham tube for gas detection. The results were interpreted according to [17]. Uninoculated MB medium with an inverted Durham tube served as a control. Carbohydrate utilization (0.5%, *w*/*v*) as sole carbon and energy sources was determined in basal medium [20] at 20 °C for 14 days. Growth was evaluated by measuring the optical density at 600 nm (OD_600_). Growth was scored as positive (+) when the OD_600_ was at least twofold that of the negative control (without any carbon source), weakly positive (w) when the OD_600_ was more than 1.5-fold but no more than twofold that of the negative control, and negative (−) when the OD_600_ was less than or equal to 1.5-fold that of the negative control. Antibiotic susceptibility was determined using the disc-diffusion method on MA plates spread with cell suspensions adjusted to 0.5 McFarland standard. Ten antibiotic discs (Oxoid, Basingstoke, UK) were tested. Inhibition zone diameters were measured after 5 days at 20 °C. A clear zone larger than 10 mm (including the 6 mm disc diameter) was recorded as sensitive [21]. Anaerobic growth was evaluated in MB supplemented with 0.1% (*w*/*v*) KNO_3_, 0.05% (*w*/*v*) L-cysteine hydrochloride and 0.05% (*w*/*v*) Na_2_S in Hungate tubes filled with oxygen-free N_2_, incubated at 20 °C for two weeks. Additional biochemical characteristics were determined with API ZYM and API 20NE strips (bioMérieux, Marcy-l’Étoile, France) following the manufacturer’s instructions. Sterile artificial seawater (ASW) [6] was used as the cell suspension solution, and the AUX medium was supplemented with 2.0% (*w*/*v*) NaCl. Strips were incubated at 20 °C and recorded after 24 h (API ZYM) or 48 h (API 20NE).

For cellular fatty acid analysis, cells were harvested from the third quadrant of MA plates after incubation at 20 °C under standardized growth conditions. Fatty acid methyl esters were separated and identified using an Agilent 6850N gas chromatograph coupled (Agilent Technologies, Inc., Santa Clara, CA, USA) with the Sherlock microbial identification system (TSBA library, version 6.1; MIDI, Newark, DE, USA). The analysis was performed at the Yellow Sea Fisheries Research Institute, Chinese Academy of Fishery Sciences (Qingdao, China). For polar lipid and respiratory quinone analysis, cells were grown in MB at 20 °C to early stationary phase. Polar lipids were extracted following the protocol of [22] and separated by two-dimensional TLC on silica gel 60 F_254_ plates (Merck, Darmstadt, Germany). Spots were visualized by spraying with ethanolic molybdophosphoric acid (for total lipids), ninhydrin (for aminolipids), and molybdenum blue (for phospholipids). Respiratory quinones were extracted, purified, and analyzed as described by [23].

## 3. Results and Discussion

### 3.1. 16S rRNA Gene Phylogeny

**Figure 1:**
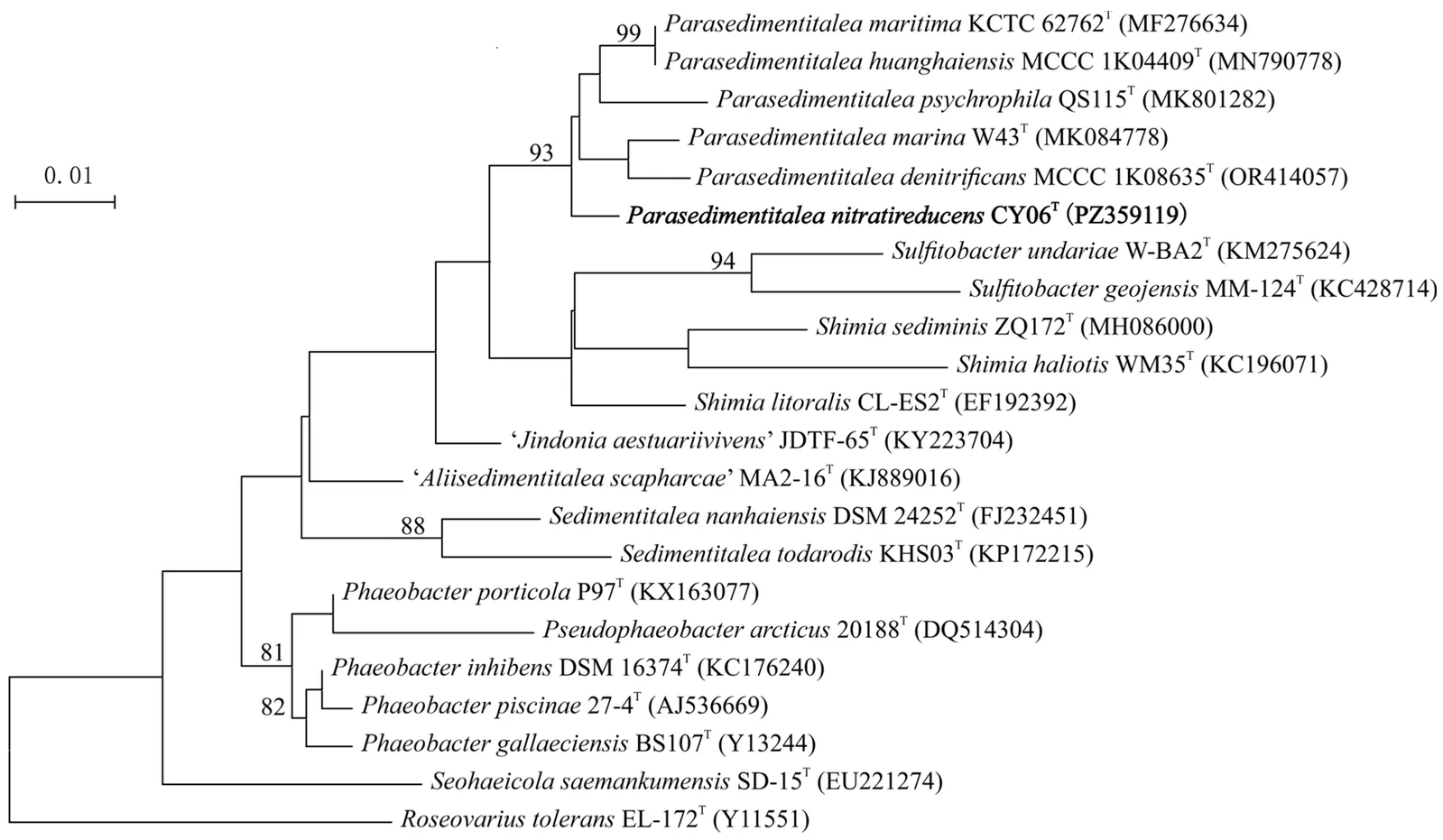
Maximum-likelihood phylogenetic tree based on 16S rRNA gene sequences showing the position of strain CY06^T^ (in bold) among related type strains. *Roseovarius tolerans* EL-172^T^ was used as an outgroup. Bootstrap values (≥70%) based on 1000 replicates are shown at nodes. Scale bar, 0.01 substitutions per nucleotide position.

**Figure 2:**
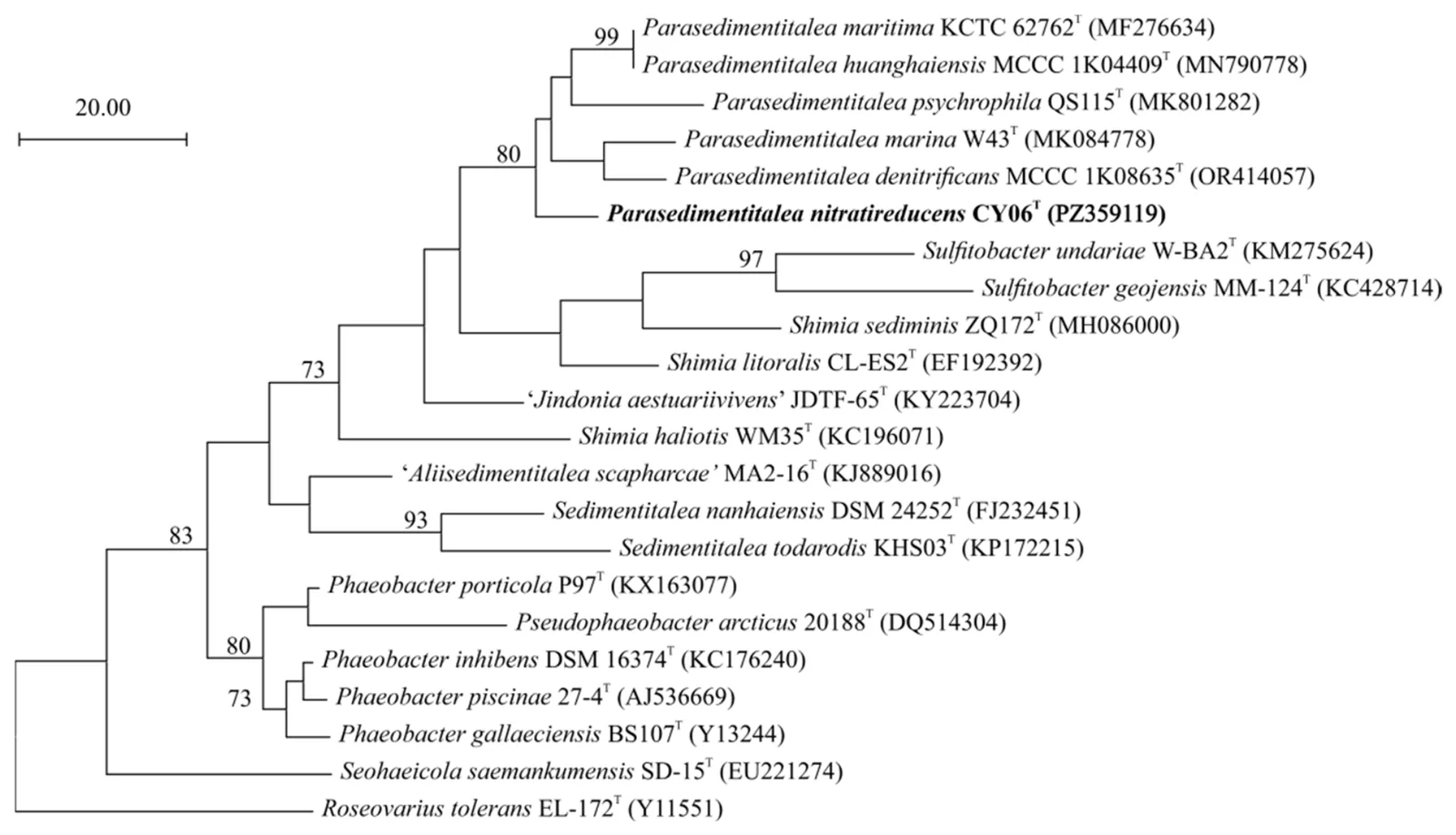
Maximum-parsimony phylogenetic tree based on 16S rRNA gene sequences showing the phylogenetic positions of strains CY06^T^ (in bold) and related type strains. *Roseovarius tolerans* EL-172^T^ was used as an outgroup. Bootstrap values (≥70%) based on 1000 replicates are shown at nodes.

**Figure 3:**
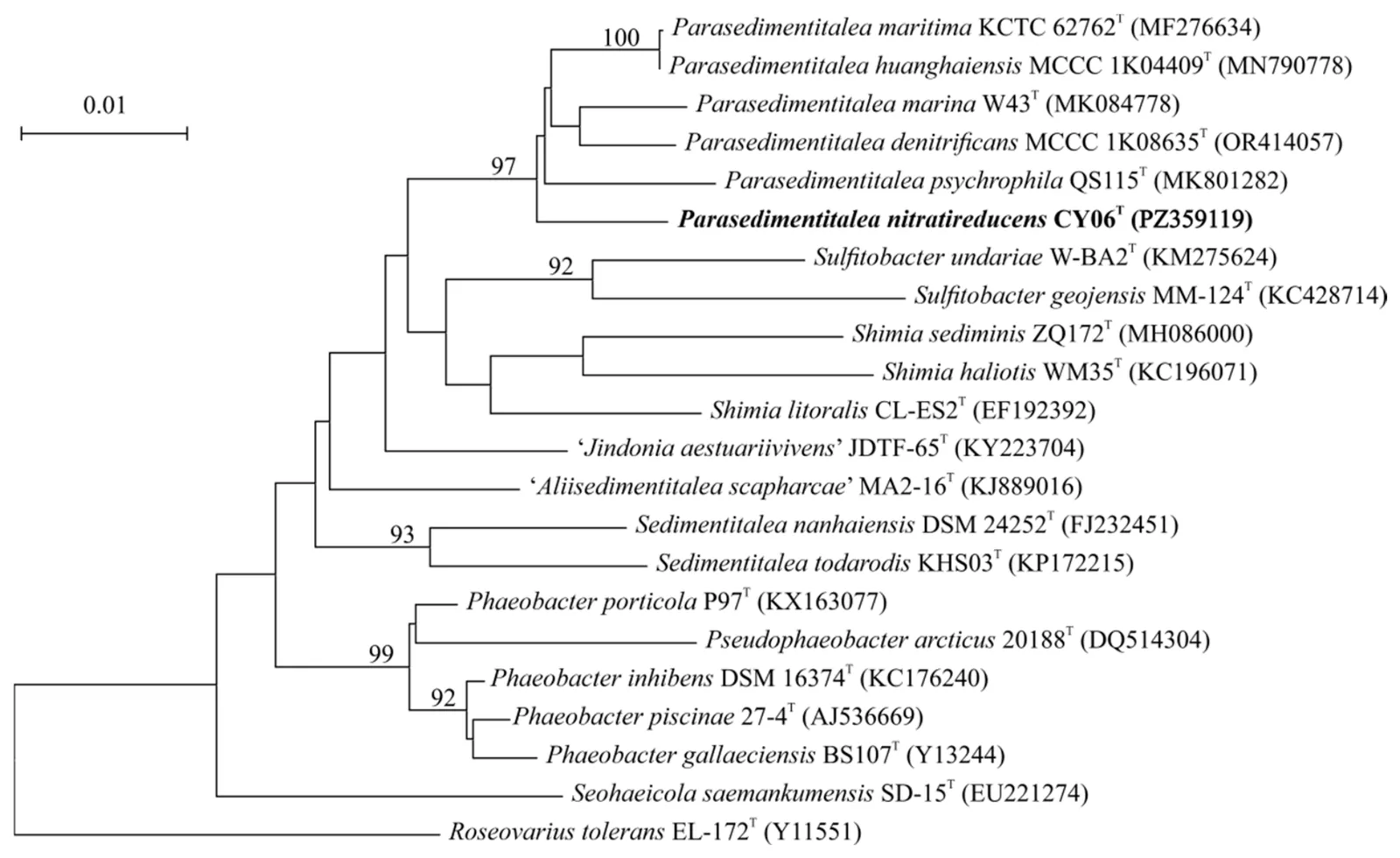
Neighbour-joining phylogenetic tree based on 16S rRNA gene sequences showing the phylogenetic positions of strains CY06^T^ (in bold) and related type strains. *Roseovarius tolerans* EL-172^T^ was used as an outgroup. Bootstrap values (≥70%) based on 1000 replicates are shown at nodes.

The nearly full-length 16S rRNA gene sequence (1425 bp, GenBank accession No. PZ359119) of strain CY06^T^ was obtained. Based on comparisons of 16S rRNA gene sequences, strain CY06^T^ showed the highest similarity to *P. huanghaiensis* MCCC 1K04409^T^ (98.56%) and *P. maritima* KCTC 62762^T^ (98.56%), followed by *P. denitrificans* MCCC 1K08635^T^ (98.27%), *P. marina* W43^T^ (98.11%), and *P. psychrophila* QS115^T^ (97.91%). Sequence similarities to the type strains of other closely related members of the family *Roseobacteraceae* were below 97.10%, which is lower than the proposed threshold (98.70%) for novel species delineation [24]. In the maximum-likelihood tree (Figure 1), CY06^T^ fell within the clade comprising members of the genus *Parasedimentitalea* with 93.0% bootstrap support. This stable topology was also supported by the maximum-parsimony (Figure 2) and neighbor-joining trees (Figure 3). Taken together, these results indicate that strain CY06^T^ represents a novel species of the genus *Parasedimentitalea*.

### 3.2. Genome Features and Genome-Based Phylogeny

The assembled draft genome of strain CY06^T^ was 5.0 Mb in length, with 48 contigs and an N50 value of 599.6 Kb. The genomic DNA G+C content of strain CY06^T^ was 55.5%, which is similar to that of its close relatives (Table 1).

**Figure 4:**
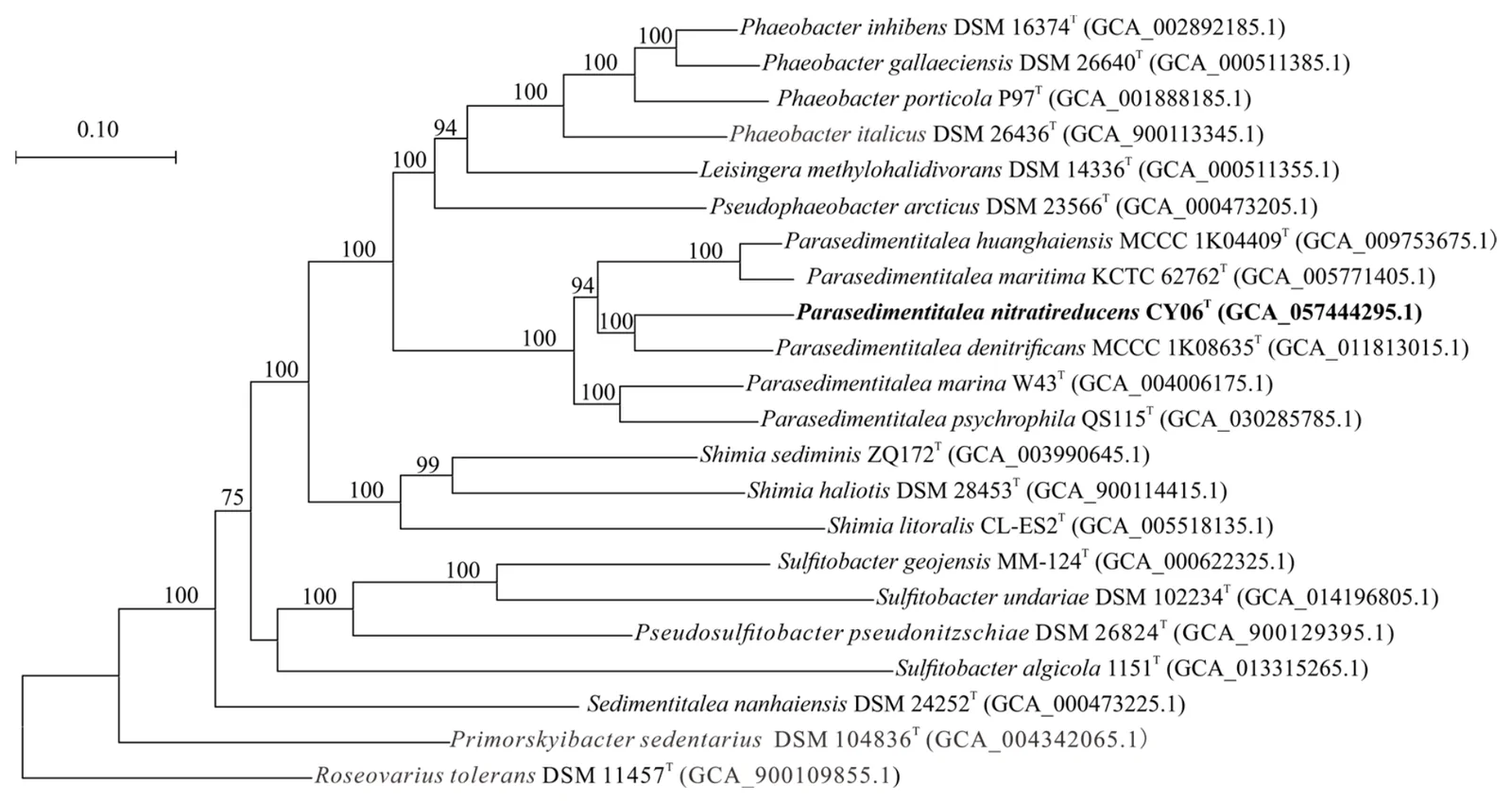
UBCG-based maximum-likelihood phylogenomic tree constructed from concatenated sequences of 92 universal bacterial core genes. Strain CY06^T^ is shown in bold. Bootstrap values (≥70%; 100 replicates) are shown at nodes. *Roseovarius tolerans* EL-172^T^ was used as an outgroup. Scale bar, 0.1 substitutions per position.

To complement the phylogenetic analyses based on the 16S rRNA gene, a genome-based phylogeny was reconstructed using the UBCG tool. In the genome-based tree (Figure 4), strain CY06^T^ first formed a well-supported subclade with *P. denitrificans* MCCC 1K08635^T^ (100% bootstrap support). This subclade was then clustered with other type species of the genus *Parasedimentitalea* (also 100% bootstrap support). Together with the 16S rRNA gene trees, the phylogenomic evidence firmly confirms that strain CY06^T^ represents a novel species within the genus.

Further, genome-based similarity indices, including dDDH, ANIb and AAI, which are widely used in prokaryotic taxonomy, were calculated (Table 2). The dDDH values between strain CY06^T^ and *P. huanghaiensis* MCCC 1K04409^T^, *P. maritima* KCTC 62762^T^, and *P. denitrificans* MCCC 1K08635^T^ were 22.00%, 22.30% and 23.70%, respectively, all well below the recommended threshold (70%) for species demarcation [25]. Similarly, the ANIb values between strain CY06^T^ and the same three reference strains were 77.85%, 77.96% and 79.50%, respectively, which were all significantly lower than the widely suggested acceptable species threshold (95.0–96.0%) [26,27]. The AAI values between strain CY06^T^ and the same three reference strains were 84.30%, 84.24% and 85.41%, respectively, which are also well below the proposed cut-off (≥95%) for species delineation [28].

Together, the genome-based phylogenetic analysis and genomic relatedness indices (dDDH, ANIb and AAI) strongly support the conclusion that strain CY06^T^ represents a novel species of the genus *Parasedimentitalea*, corroborating the phylogenetic evidence obtained from 16S rRNA gene sequences.

### 3.3. Phenotypic and Chemotaxonomic Characterizations

**Figure 5:**
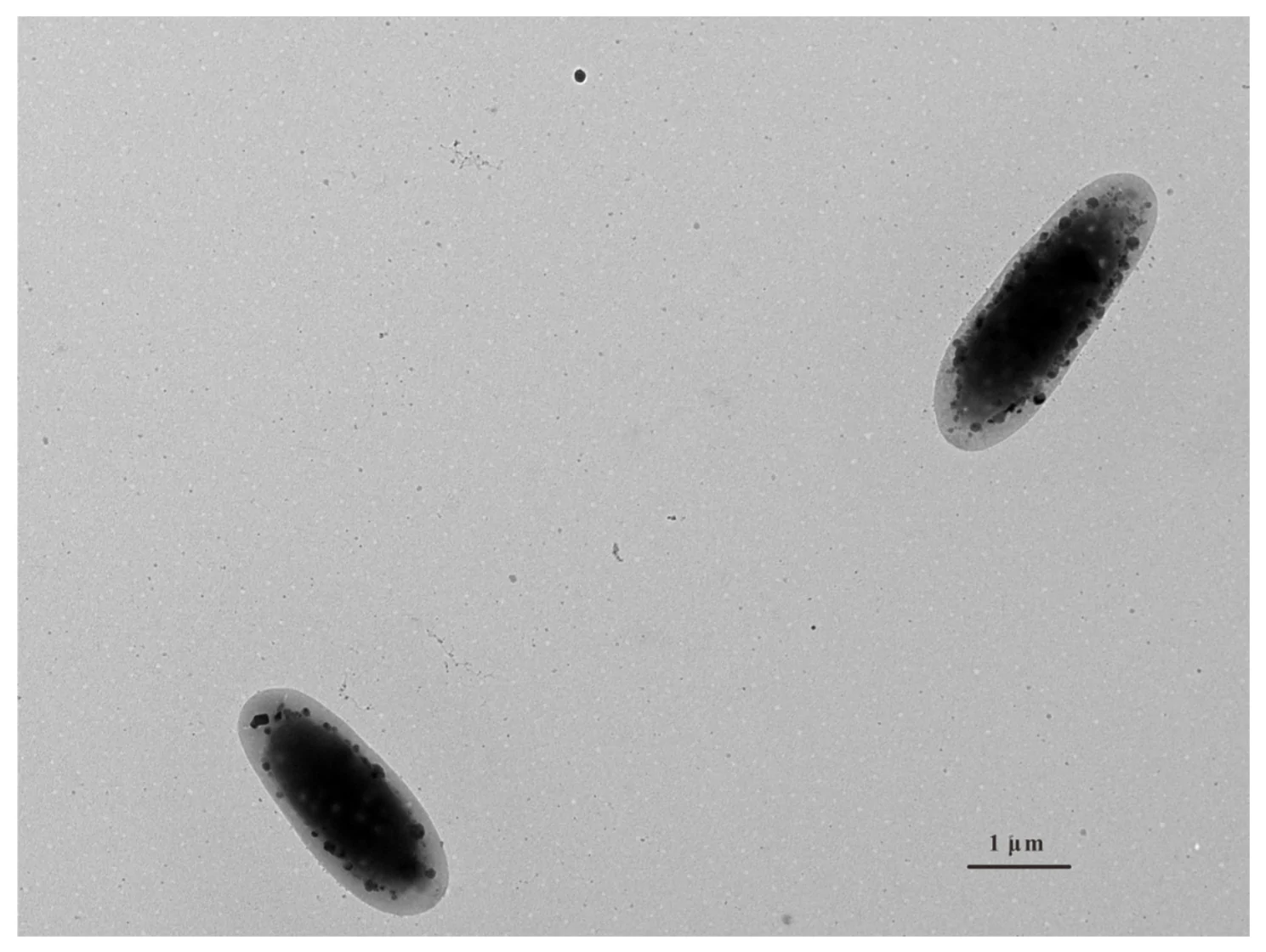
Transmission electron micrograph showing the morphology of strain CY06^T^ incubated on MA (20 °C, 4 days). No flagella were observed. Scale bar, 1.0 μm.

Strain CY06^T^ is aerobic, rod-shaped, and approximately 0.9–1.3 μm in width and 1.1–3.2 μm in length (Figure 5). No swimming motility was observed by light microscopy using wet mounts, and no flagella were detected by transmission electron microscopy or flagella staining. Colonies are circular with regular edges, opaque, slightly convex, and approximately 0.2–2.0 mm in diameter on marine agar after 7 days of incubation at 20 °C. Growth occurs at 5–30 °C (optimum, 20 °C), pH 6.5–9.0 (optimum, pH 7.0–7.5), and with 1.0–5.0% (*w*/*v*) NaCl (optimum, 2.0–3.0%), but no growth occurs in the absence of NaCl.

Strain CY06^T^ was sensitive to bacitracin (10 U), cefotaxime (30 μg), chloramphenicol (30 μg), nitrofurantoin (300 μg), penicillin G (10 U) and rifampicin (5 μg), but resistant to amoxycillin (10 μg), carbenicillin (100 μg), ampicillin (10 μg) and cefoxitin (30 μg). The complete API ZYM and API 20NE reaction profiles of strain CY06^T^ were determined and are summarized as follows. In the API ZYM test, the strain was positive for alkaline phosphatase, esterase (C4), esterase lipase (C8), leucine arylamidase, valine arylamidase, acid phosphatase and naphthol-AS-BI-phosphohydrolase, but negative for lipase (C14), cystine arylamidase, trypsin, α-chymotrypsin, α-galactosidase, β-galactosidase, β-glucuronidase, α-glucosidase, β-glucosidase, N-acetyl-β-glucosaminidase, α-mannosidase and α-fucosidase. In the API 20NE test, strain CY06^T^ was positive only for nitrate reduction and negative for all other reactions tested, including indole production, acid production from glucose, arginine dihydrolase, urease, β-glucosidase, gelatinase and β-galactosidase, as well as assimilation of glucose, arabinose, mannose, mannitol, N-acetyl-glucosamine, maltose, gluconate, caprate, adipate, malate, citrate and phenylacetate.

The nitrate reduction, nitrite reduction and denitrification abilities of strain CY06^T^ were further assessed using MB medium supplemented with KNO_3_ or NaNO_2_. Strain CY06^T^ reduced nitrate to nitrite but did not reduce nitrite further, and no gas production was observed. To provide genomic support for the observed nitrate reduction phenotype and the absence of denitrification, genes associated with nitrate reduction and denitrification were examined in the annotated genome of strain CY06^T^. The *napEFDABC* gene cluster (locus_tags: *AC2T28_RS12660*, *AC2T28_RS12665*, *AC2T28_RS12670*, *AC2T28_RS12675*, *AC2T28_RS12680* and *AC2T28_RS12685*), encoding components of a putative periplasmic nitrate reductase system, was identified, which is consistent with the observed ability of strain CY06^T^ to reduce nitrate to nitrite [29]. In contrast, no complete set of genes required for denitrification was identified. Genes encoding the key denitrification enzymes responsible for nitrite reduction, including the cytochrome *cd*_1_-type nitrite reductase (nirS) and the copper-containing nitrite reductase (nirK), were absent [30]. Likewise, no *norBC* gene cluster encoding nitric oxide reductase was identified. Within the *nos* gene cluster, *nosZ* (locus_tag: *AC2T28_RS18385*) together with the accessory genes *nosD* (locus_tag: *AC2T28_RS18390*) and *nosL* (locus_tag: *AC2T28_RS18405*) was detected, whereas the remaining genes required for a complete nitrous oxide reductase system were absent. These genomic features are consistent with the physiological characteristics of strain CY06^T^, indicating that strain CY06^T^ is capable of reducing nitrate to nitrite but lacks the genetic capacity for complete denitrification.

The chemotaxonomic characteristics were consistent with the phylogenetic placement of strain CY06^T^. The cellular fatty acid compositions of strain CY06^T^ and the type strains of closely related species are shown in Table 3. The predominant cellular fatty acids (>10%) of strain CY06^T^ were C_18:1_ *ω*7c and/or C_18:1_ *ω*6c, which are characteristic of the genus *Parasedimentitalea*. The major respiratory quinone is ubiquinone-10. The polar lipid profile consists of phosphatidylcholine (PC), phosphatidylglycerol (PG), phosphatidylethanolamine (PE), an unidentified aminolipid (AL), an unidentified lipid (L) and three unidentified phospholipids (PL) (Figure S1).

Collectively, these phenotypic, chemotaxonomic and genomic characteristics further distinguish strain CY06^T^ from its closest relatives and support its recognition as representing a novel species of the genus *Parasedimentitalea*.

## 4. Conclusions

Strain CY06^T^ exhibited clear phylogenetic, genomic, phenotypic and chemotaxonomic differences from its closest relatives. The phylogenetic analyses based on the 16S rRNA gene and genome-scale core genes, together with genome relatedness indices (dDDH, ANIb and AAI), as well as differential physiological and chemotaxonomic characteristics, consistently supported its classification as a distinct species within the genus *Parasedimentitalea*. Therefore, based on the polyphasic evidence presented in this study, strain CY06^T^ represents a novel species of the genus *Parasedimentitalea*, for which the name *Parasedimentitalea nitratireducens* sp. nov. is proposed.

### Description of Parasedimentitalea nitratireducens *sp. nov.*

*Parasedimentitalea nitratireducens* [ni.tra.ti.re.du’cens. N.L. neut. n. *nitras* (gen. nitratis), nitrate; L. pres. part. *reducens*, converting to a different state; N.L. part. adj. nitratireducens, reducing nitrate (to nitrite)].

Cell of CY06^T^ are Gram-stain-negative, aerobic, rod-shaped, non-flagellated, and approximately 0.9–1.3 μm in width and 1.1–3.2 μm in length. Colonies on marine agar after incubation for 7 days at 20 °C were circular with regular edges, opaque, slightly convex, and approximately 0.2–2.0 mm in diameter. Growth occurs at 5–30 °C (optimum, 20 °C) and at pH 6.5–9.0 (optimum, pH 7.0–7.5). Growth occurs in the presence of 1.0–5.0% (*w*/*v*) NaCl (optimum, 2.0–3.0%), but no growth occurs in the absence of NaCl. Positive for catalase and oxidase and reduced nitrate to nitrite without further denitrification. Negative for hydrolysis of Tween 40, Tween 60 and Tween 80. The following substrates were utilized as sole carbon sources for growth: D-glucose, sucrose, D-cellobiose, inositol, inulin, D-raffinose pentahydrate, D-galactose, D-mannose, D-maltose monohydrate, D-xylose (weak), xylan (weak), D-fructose (weak), D-mannitol (weak), sorbitol (weak) and D-trehalose (weak). No growth was observed with xylitol, L-sorbose or L-arabinose.

The predominant cellular fatty acids (>10%) are C_18:1_ *ω7c* and/or C_18:1_ *ω6c*. The major respiratory quinone is ubiquinone-10. The polar lipid profile consists of phosphatidylcholine, phosphatidylglycerol, phosphatidylethanolamine, an unidentified aminolipid, an unidentified lipid and three unidentified phospholipids.

The type strain is CY06^T^ (=MCCC 1K08636^T^ = KCTC 72006^T^), isolated from surface sediment of the Yellow Sea. The genomic DNA G+C content is 55.5%, and the approximate genome size is 5.0 Mb. The GenBank accession numbers for the 16S rRNA gene sequence and the draft genome sequence of the type strain are PZ359119 and JBYAXC000000000, respectively.

## Figures and Tables

**Table 1:** Differential characteristics between strain CY06^T^ and the closely related type species of the genus *Parasedimentitalea*. Strains: 1, CY06^T^; 2, *P*. *huanghaiensis* MCCC 1K04409^T^; 3, *P*. *maritima* KCTC 62762^T^; 4, *P*. *denitrificans* MCCC 1K08635^T^; 5, *P. marina* W43^T^; 6, *P. psychrophila* QS115^T^. Unless otherwise indicated, all data were obtained in this study. Data for strains *P. marina* W43^T^ and *P. psychrophila* QS115^T^ were taken from [1] and [2], respectively. Data indicated by superscripts were taken from: a, [5]; b, [4]; c, [3]. +, positive; −, negative; w, weakly positive; nr, not reported.

Characteristic	1	2	3	4	5	6
Cell morphology	rods	rods or ovoid-shaped rods ^a^	rods ^b^	rods ^c^	rod-shaped	rod-shaped
Reduce nitrate to nitrite	+	+	+	+	−	–
Reduction of nitrite	−	+	+	+	nr	nr
Denitrification	−	+	+	+	−	–
Growth at/with:						
5 °C	+	+	+	−	+	+
pH 9.5	−	−	−	+	−	–
6% (*w*/*v*) NaCl	−	−	+	+	+	–
Assimilation of:						
D-glucose	+	+	−	+	nr	nr
Sucrose	+	+	−	−	nr	nr
D-Cellobiose	+	−	−	+	nr	nr
Inulin	+	+	w	−	nr	nr
Inositol	+	−	−	+	nr	nr
G+C content (%)	55.5	54.2 ^a^	54.3 ^b^	55.0 ^c^	55.9	57.7
Isolation source	marine sediment	marine sediment ^a^	deep-sea water ^b^	marine sediment ^c^	deep-sea water	deep-sea sediment

**Table 2:** 16S rRNA gene sequence similarities and genome-based indices (dDDH, ANIb and AAI) between strain CY06^T^ and the type strains of species of the genus *Parasedimentitalea*.

Species (Assembly Accession)	16S Similarity	dDDH	ANIb	AAI
*P. huanghaiensis* MCCC 1K04409^T^ (GCA_009753675.1)	98.56%	22.00%	77.85%	84.30%
*P. maritima* KCTC 62762^T^ (GCA_005771405.1)	98.56%	22.30%	77.96%	84.24%
*P. denitrificans* MCCC 1K08635^T^ (GCA_011813015.1)	98.27%	23.70%	79.50%	85.41%
*P. marina* W43^T^ (GCA_004006175.1)	98.12%	21.30%	77.22%	82.65%
*P. psychrophila* QS115^T^ (GCA_030285785.1)	97.91%	21.50%	77.60%	82.39%

**Table 3:** Cellular fatty acid compositions (%) of strain CY06^T^ and closely related type strains. Strains: 1, CY06^T^; 2, *P*. *huanghaiensis* MCCC 1K04409^T^; 3, *P*. *maritima* KCTC 62762^T^; 4, *P*. *denitrificans* MCCC 1K08635^T^. All data were obtained from this study. Values below 0.2% for any of the four strains are not shown. −, not detected. The major fatty acids (>10%) of strain CY06^T^ are highlighted in bold.

Fatty Acid	1	2	3	4
C_12:0_	0.14	0.20	0.19	0.28
C_16:0_	4.15	6.10	1.42	2.35
C_18:0_	0.88	1.43	0.84	2.16
C_10:0_ 3OH	4.79	6.68	2.50	3.12
C_16:0_ 2OH	4.90	4.28	2.43	1.70
C_16:1_ 2OH	−	0.40	0.26	−
C_12:1_ 3OH	−	0.84	−	−
C_18:1_ 2OH	0.83	1.30	1.11	0.51
C_16:1_ *ω*7*c* and/or C_16:1_ *ω*6*c*	0.49	−	−	−
**C_18:1_ ** ***ω*** **7** ***c*** **and/or C_18:1_ ** ***ω*** **6** ***c***	**83.83**	**78.62**	**90.64**	**89.20**

## Data Availability

The 16S rRNA gene sequence of strain CY06^T^ is publicly available in the GenBank database under accession number PZ359119. The Whole Genome Shotgun project is publicly available in DDBJ/ENA/GenBank under accession number JBYAXC000000000. The version described in this paper is version JBYAXC010000000.

## Supplementary Materials

The following supporting information can be downloaded at: https://www.mdpi.com/article/10.3390/microorganisms14081706/s1, Figure S1: Polar lipid profile of strain CY06^T^. (a) Total lipids stained with ethanolic molybdophosphoric acid; (b) phospholipids stained with molybdenum blue; (c) aminolipids stained with ninhydrin. PC, phosphatidylcholine; PG, phosphatidylglycerol; PE, phosphatidylethanolamine; L, unidentified lipid; PL, unidentified phospholipid; AL, unidentified aminolipid.

## Abbreviations

The following abbreviations are used in this manuscript:

MA	Marine agar 2216
MB	Marine broth 2216
dDDH	Digital DNA-DNA hybridization
ANI	Average nucleotide identity
AAI	Average amino acid identity
PC	phosphatidylcholine
PG	phosphatidylglycerol
PE	phosphatidylethanolamine
AL	unidentified aminolipid
L	unidentified lipid
PL	unidentified phospholipid
