# Supplementary material for: Parasedimentitalea nitratireducens sp. nov., a Novel Nitrate-Reducing Bacterium Isolated from the Yellow Sea
Source: Microorganisms. 2026 Aug 4;14(8):1706. doi: 10.3390/microorganisms14081706 (PMC13515762; doi:10.3390/microorganisms14081706)
Supplement: Supplementary file 1 [file microorganisms-14-01706-s001.zip › microorganisms-4335297-supplementary.pdf]

Supplementary materials

Parasedimentitalea nitratireducens sp. nov., a Novel Nitrate-Reducing Bacterium Isolated from the Yellow Sea

Yanjiao Zhang, Xinyu Liu, Hao Yu and Xiuqing Yang *

Shandong Provincial Key Laboratory of Microbial Resource Exploration and Innovative Utilization, School of Life Sciences, Qingdao Agricultural University, Qingdao 266109, China; zhangyanjiao34@163.com (Y.Z.); 17664422874@163.com (X.L.); yuhaosunshine@163.com (H.Y.)

* Correspondence: qdndyxq@163.com

Supplementary figure legend

Figure S1. Polar lipid profile of strain CY06^T. (a) Total lipids stained with ethanolic molybdophosphoric acid; (b) phospholipids stained with molybdenum blue; (c) aminolipids stained with ninhydrin. PC, phosphatidylcholine; PG, phosphatidylglycerol; PE, phosphatidylethanolamine; L, unidentified lipid; PL, unidentified phospholipid; AL, unidentified aminolipid.

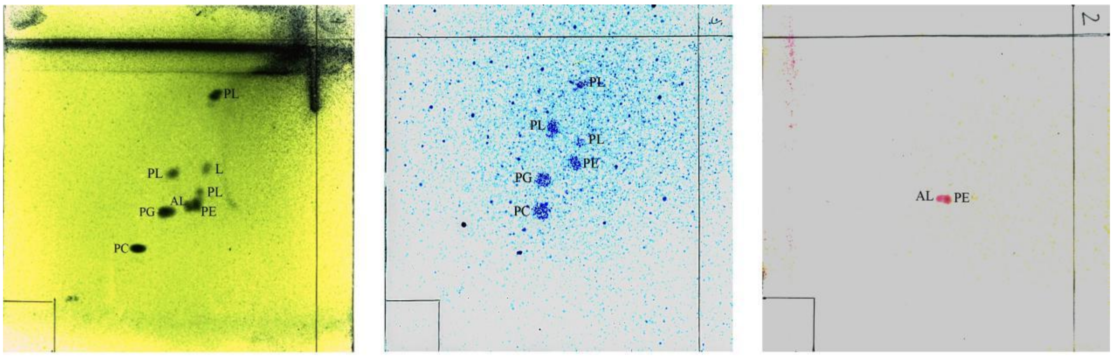

(a) (b) (c)
